# Dysregulated miRNA Expression and Androgen Receptor Loss in Racially Distinct Triple-Negative Breast Cancer

**DOI:** 10.3390/ijms252413679

**Published:** 2024-12-21

**Authors:** Shristi Bhattarai, Bruna M. Sugita, Emanuelle Nunes-Souza, Aline S. Fonseca, Darshan Shimoga Chandrashekar, Mahak Bhargava, Luciane R. Cavalli, Ritu Aneja

**Affiliations:** 1 Department of Molecular and Cellular Biology, Kennesaw State University, Kennesaw, GA 30144, USA; 2 Department of Biology, Georgia State University, Atlanta, GA 30302, USA; 3 Research Institute Pelé Pequeno Príncipe, Faculdades Pequeno Príncipe, Curitiba 80250-060, PR, Brazil; brunasugita@yahoo.com (B.M.S.);; 4 Molecular and Cellular Pathology, Department of Pathology, University of Alabama at Birmingham, Birmingham, AL 35233, USA; dshimogachandrasheka@uabmc.edu; 5 Department of Nutrition Sciences, School of Health Professions, University of Alabama at Birmingham, Birmingham, AL 35294, USA; 6 Department of Oncology, Lombardi Comprehensive Cancer Center, Georgetown University, Washington, DC 20007, USA

**Keywords:** miRNA, triple-negative breast cancer, quadruple negative, race, androgen receptor, prognosis

## Abstract

Androgen receptor (AR)-negative triple-negative breast cancer (TNBC), often termed quadruple-negative breast cancer (QNBC), disproportionately impacts women of African descent, leading to poorer overall survival (OS). MiRNAs regulate the expression of gene drivers involved in critical signaling pathways in TNBC, such as the *AR* gene, and their expression varies across races and breast cancer subtypes. This study investigates whether differentially expressed miRNAs influence AR transcription, potentially contributing to the observed disparities between African American (AA) and European American (EA) QNBC patients. Race-annotated TNBC samples (n = 129) were analyzed for AR expression status and revealed the prevalence of QNBC in AA patients compared to EA (76.6% vs. 57.7%) and a significant association of AR loss with poor survival among AAs. The Cancer Genome Atlas (TCGA) RNA-seq data showed that AAs with TNBC (n = 32) had lower AR mRNA levels than EAs (n = 67). Among TCGA patients in the AR-low group, AAs had significantly poorer OS than EAs. In our cohort, 46 miRNAs exhibited differential expression between AAs and EAs with QNBC. Ten of these miRNAs (miR-1185-5p, miR-1305, miR-3161, miR-3690, miR-494-3p, miR-509-3-5p, miR-619-3p, miR-628-3p, miR-873-5p, and miR-877-5p) were predicted to target the AR gene/signaling. The loss of AR expression is linked to poorer prognoses in AA women. The understanding of the specific miRNAs involved and their regulatory mechanisms on AR expression could provide valuable insights into why AA women are more prone to QNBC.

## 1. Introduction

Triple negative breast cancer (TNBC) is a heterogenous disease classified into major molecular subtypes [basal-like1, basal-like2, mesenchymal, and luminal androgen receptor (LAR)], each characterized by different clinicopathologic and molecular features [1]. TNBC prevalence is higher in African American (AA) women than in European American (EA) women after adjusting for confounding variables [2]. AA women are 2–3 times more likely to develop TNBC than EA women. Many studies suggest that among TNBC patients, AAs still experience worse clinical outcomes than EAs [3]. AAs are diagnosed at a younger age and exhibit larger tumor size, faster tumor proliferation, and higher intratumoral heterogeneity compared to EAs among TNBC patients [4]. Furthermore, the increased up-regulation of Ki-67, C-Kit, ALDH1, IGFR, VEGR and loss of BRCA1 expression among AA compared to EA TNBC tumors has been speculated to underlie the racial disparate burden in TNBCs [5,6,7,8,9]. Recently, higher TNBC incidence and severity was reported among West Africans compared to AAs and other ethnic groups [10]. AAs share ancestry with native West Africans, which may rationalize greater incidence and severity of TNBC among AAs compared to EAs [11]. Notably, TNBC accounts for 50–70% of breast cancers (BCs) in Nigerian [12], 80% in Ghanaian [13] and 30% in AA women, but only 15% in EA [13,14] or white British women [12,14]. Distinctions in inherent tumor biology have been suggested as a potential explanation for the global racial disparities in TNBC burden.

Androgen receptor (AR) status subclassifies triple-negative breast cancer (TNBC) into AR-positive TNBC (AR+ TNBC) and AR-negative TNBC or quadruple-negative breast cancer (AR− or QNBC). AR+ TNBC is more common among EAs, usually expresses low levels of basal biomarkers, and is sensitive to AR antagonists [15,16]. Conversely, AAs commonly harbor the highly proliferative and aggressive basal-like subtype lacking AR expression and are mostly non-responsive to AR antagonists [16]. The prognostic value of AR expression in TNBC is ambiguous because of the lack of consideration of patients’ biogeographic ancestry and other population-specific factors and the underrepresentation of AAs in US cohorts [17]. Our previous studies have highlighted that TNBC patients of African descent lack AR (80% of AAs, 90% Africans vs. 45% EAs) and that AA QNBC patients have worse overall survival than EAs [16,17]. Moreover, comprehensive assessments of androgen receptor (AR) expression across all breast cancer subtypes have shown that AA women are more likely to lack AR expression, with the highest frequency of AR loss seen in TNBC. Among AA women with AR-negative TNBC, the overall survival rates were worse compared to EA women [18]. QNBC landscape is distinct from TNBC, and AA QNBCs exhibit a phenotype distinct from EA QNBC. The distinct molecular profile of QNBC deems its classification as a clinically relevant, independent subtype, instead of its inclusion within TNBC, and warrants a thorough characterization. The glaring racial disparity is not just confined to the prevalence and molecular portrait of this intractable disease, but spills over to treatment options as well, for the AA demographic. AR antagonists have shown promise in TNBC patients, but no targeted drugs are available or under development for QNBC. QNBC patients, who are predominantly AAs, currently have no treatment options beyond chemotherapy.

MiRNAs, small non-coding RNAs, are attractive therapeutic targets for TNBC patients owing to their inherent ability to regulate expression of multiple altered mRNAs that underlie disease progression. Through binding to their mRNA targets, miRNAs can regulate cancer-related gene networks and signaling pathways, impacting TNBC biological phenotype and clinical outcomes [19,20]. A plethora of studies have identified dysregulated expression levels of miRNAs unique to TNBC development and progression and have uncovered a prognostic or predictive role for these miRNAs in the disease. The deregulated expression of specific miRNA clusters, signatures, and individual miRNAs in TNBC are associated with oncogenic pathways such as Epithelial–Mesenchymal Transition (EMT), PI3K/Akt/mTOR, MYC, and PTEN. In addition, it has been associated with proliferation, migration, invasion, and chemoresistance, as well as with metastasis development, relapse, and poorer overall survival (OS) [21]. MiRNA expression also varies by race or ethnicity [22]. In AA patients, miRNA polymorphisms are associated with a higher BC susceptibility [23,24,25,26,27]; however, there is a lack of data on somatic miRNA expression in AA tumors. Few studies have shown variation in miRNA expression in TNBC tissues of AAs vs. EAs or other race or ethnic groups [28,29,30,31,32,33,34]. Some of our own studies showed that there is a differential tumor miRNA expression pattern in genomically characterized AAs vs. EAs with TNBCs [32,33].

In QNBC, a previous study reported 153 miRNAs differentially expressed compared to AR-positive molecular subtypes. These miRNAs, through the regulation of their target genes, affected several signaling pathways involved in tumor cell proliferation and invasion [35]. Another study using TCGA data identified 45 miRNAs that were differentially clustered by AR status and subtype [36]. In our previous study on QNBCs, we showed 184 differentially expressed miRNAs when compared to TNBCs. Eight of these miRNAs (miR-1204, miR-1265, miR-1267, miR-23c, miR-548ai, miR-567, miR-613, and miR-943) presented concordance of expression and copy number levels, and robustly discriminated TNBCs from QNBCs [37]. In this study, our aim is to determine whether the loss of AR expression in AA QNBCs results from miRNA regulation in QNBC patients and underlies disparity in QNBCs, improving the understanding of racial differences in breast cancer outcomes.

## 2. Results

### 2.1. Loss of AR Associated with AA TNBCs

**Figure 1:**
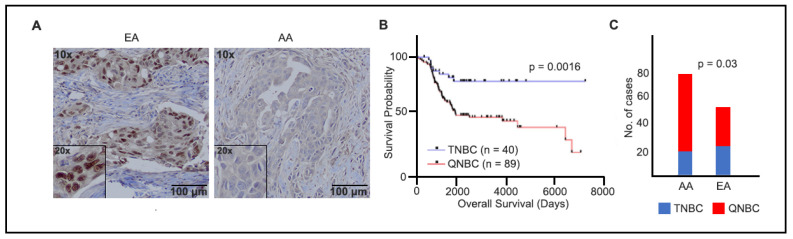
AR immunostaining and overall survival analysis in TNBC samples of EA and AA patients. (**A**) Micrographs representing AR staining in tumor tissues from EAs (80%) and AAs (6%), AR (brown) and nuclei (blue), Insets: 20× objective; (**B**) Kaplan–Meier plots of overall survival in TNBC (n = 40) and QNBC (n = 89) patients; (**C**) cases that were EA and AA TNBC and QNBC by IHC.

To evaluate the role of AR in TNBCs, we stained 129 TNBC tissue sections, which were sourced from two institutes, as specified in the Materials and Methods section. The IHC-stained sections were independently assessed and scored by two pathologists who were blinded to the study, as shown in Figure 1A. TNBC samples displaying less than 1% AR expression were classified as AR-negative TNBCs (QNBC). We identified a significant association between QNBC status and poor overall survival (*p* = 0.0016), as depicted in Figure 1B. Our results reveal a substantial link between AA and QNBC status, with AR loss observed in 76.6% (59 out of 77) of AA tumors compared to only 57.7% (30 out of 52) of EA tumors (*p* = 0.03), as illustrated in Figure 1C.

### 2.2. Differences in AR Gene Methylation Status, Mutations, and Copy Number Alterations (CNAs) Between AA and EA QNBC

**Figure 2:**
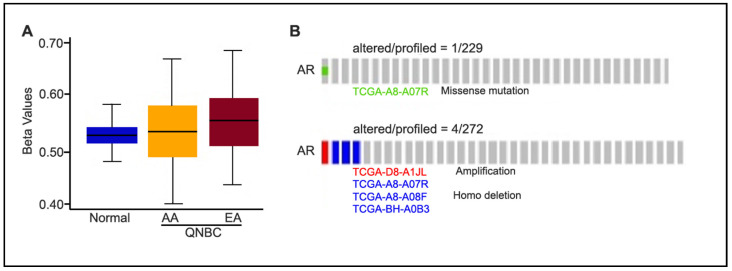
DNA methylation of *AR* gene of AA and EA patients of TCGA database. (**A**) Methylation status of *AR* promoter region in normal and QNBC (AA vs. EA). Beta value ranges from 0 to 1 (no to complete methylation); significance based on unpaired *t*-test; (**B**) oncoprint showing *AR* mutations and CNAs in AR-low samples (n = 278).

Given that AR loss was associated with AA population, we evaluated next the difference in methylation of AA and EA *AR* genes. To this end, we analyzed the TCGA BRCA dataset using built-in functions of the TCGA-assembler and calculated a single methylation value for each gene’s promoter region (−1500 to +1). We found no significant differences in the DNA methylation profiles between AA QNBC (n = 24) and EA QNBC (n = 36). Additionally, we did not observe any significant difference in DNA methylation when compared to the normal/benign cases (Figure 2A). Moreover, we also examined *AR* mutations and copy number alterations (CNAs) in AR-low samples (n = 229 for mutations, n = 272 for CNAs) using cBioPortal and found no significant mutations and CNAs in the *AR* gene (Figure 2B).

### 2.3. Differentially Expressed miRNAs Underlie Racial Disparity

**Figure 3:**
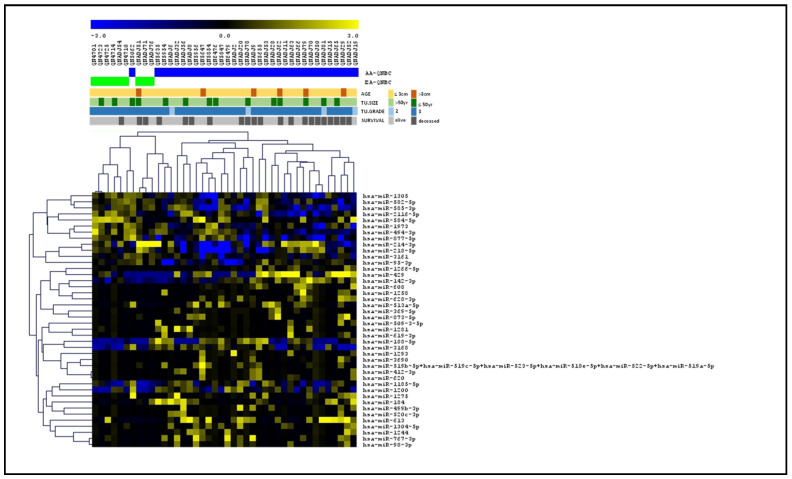
Supervised hierarchical cluster (SHC) of global miRNA expression profiling of QNBC cases of EA and AA patients. Heatmap shows 46 DE miRNAs between AA QNBC (n = 33, blue bars) and EA QNBC (n = 9, green bars) from discovery cohort. Shown below clinical data of patients [age (>50 years, 50 years), tumor size (>3 cm, ≤3 cm, and grade (2, 3), and survival status (alive, deceased)].

Our global miRNA profiling analysis (Nanostring, nCounter) comparing AA QNBCs (n = 33) and EA QNBCs (n = 9) from the Emory Decatur Hospital and Histopathology and Tissue Shared Resources (HTSR) of Lombardi Comprehensive Cancer Center (LCCC), Georgetown University) (discovery cohort) showed that 46 miRNAs were differentially expressed (DE) between AAs and EAs (Pearson correlation; *p* < 0.05, FDR < 0.05; Figure 3): 34 up-regulated and 12 down-regulated (Table S1). These miRNAs robustly clustered the samples in the two groups of patients, except for one case of an AA patient that was clustered within the EAs. Among the differentially expressed miRNAs, we found ten (10/46) miRNAs (miR-1185-5p, miR-1305, miR-3161, miR-3690, miR-494-3p, miR-509-3-5p, miR-619-3p, miR-628-3p, miR-873-5p, and miR-877-5p) predicted to target *AR* gene [six up-regulated and four down-regulated in AA QNBC vs. EA QNBC (Table 1), and seven (7/46) with experimentally validated target genes on AR signaling pathway (Table 2)]. An in-depth analysis within the TCGA BRCA dataset was conducted to investigate the relationship between AR expression (RNA-seq) and the expression of the ten miRNAs (miRNA-seq) that were predicted to target AR. First, we focused on triple-negative breast cancer (TNBC) cases within the dataset and identified eighty-one cases with available expression values for AR and for six (miR-1305, miR-3161, miR-494-3p, miR-628-3p, miR-873-5p, and miR-877-5p) of the ten miRNAs. In this TNBC subset, we observed a statistically significant positive correlation between miR-3161 and AR expression (Pearson coefficient r = 0.25, *p* < 0.05), suggesting a potential regulatory relationship. To expand our analysis, we examined all breast cancer cases in the TCGA dataset, which yielded 720 cases with expression data for both AR and the same six miRNAs. In this analysis, three miRNAs exhibited significant correlations with AR expression: miR-877-5p (r = −0.22, *p* < 0.05) and miR-873-5p (r = −0.1, *p* < 0.05) showed negative correlations with AR, while miR-628-3p presented a positive correlation (r = 0.1, *p* < 0.05).

**Figure 4:**
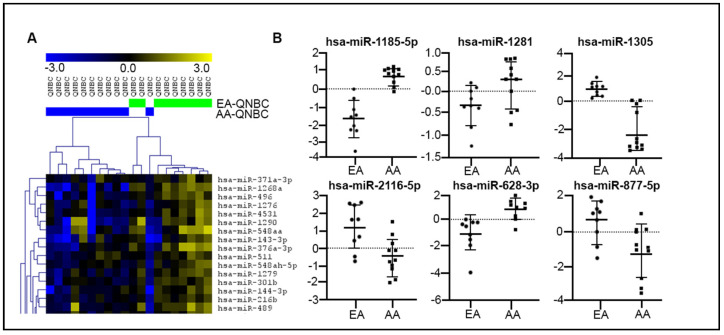
MiRNA expression profiling analysis of QNBC cases of EA and AA patients. (**A**) Partial supervised hierarchical cluster (SHC) of global miRNA profiling showing DE miRNAs between AA QNBC (n = 10, blue bars) and EA QNBC (n = 9, green bars) (validation cohort). (**B**) Six miRNAs DE between AA QNBC and EA QNBC with expression directions common in discovery and validation cohorts.

Next, we performed miRNA profiling in an independent set (validation cohort) of 19 QNBC (10 AA and 9 EA) cases from the HTSR of LCCC, Georgetown University. Within many DE miRNAs (n = 376) (Figure 4A), 14 were common to those identified in the discovery cohort (Table 3), with 6 presenting the same pattern of expression (Figure 4B). From the common 14 DE miRNAs, 5 were predicted to directly target the AR gene (miR-1185-5p, miR-1305, miR-628-3p, miR-873-5p, and miR-877-5p) and 2 targeted the AR signaling pathway (miR-1258 and miR-628-3p).

### 2.4. Six miRNAs That Discriminated AA QNBC from EA QNBC

**Figure 5:**
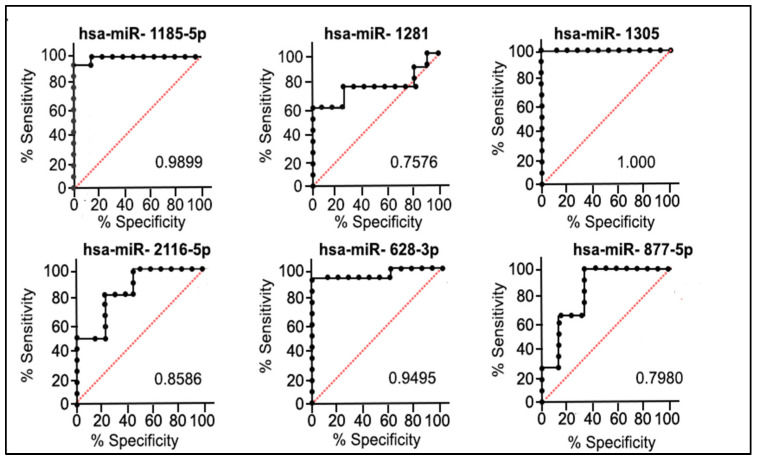
ROC analysis and corresponding *p*-values of six miRNAs (common to both discovery and validation cohorts of patients). Black line: test real classifier value; Red line: random classifier value (AUC = 0.5).

A remarkable high power was observed in the selected six miRNAs, with the same direction expression common to both the discovery and validation cohorts of the patients, in discriminating the QNBC tumors from AAs and EAs. The AUC value of the individual miRNAs ranged from 0.7576 to 1.0, with miR-1305 presenting the highest power in discriminating the two groups (Figure 5). ROC analysis was also performed with all combinations of the six selected miRNAs: a combination of three miRNAs (hsa-miR-877-5p, hsa-miR-2116-5p, and hsa-miR-1281) showed a high discriminatory potential with an AUC of 0.939.

### 2.5. Prognostic Significance of miRNA in Racially Distinct Tumors

The analysis of miRNA expression for these six specific miRNAs in relation to clinical and histopathological data was significantly correlated with race (Pearson correlation; r > 0.7, *p* < 0.05). To investigate the connection between these six miRNAs and patient survival from large datasets (TCGA and METABRIC), we employed the mirPower KMplot and gathered data from 97 TNBC samples. As illustrated in Figure S1, the lower expression levels of five of these miRNAs, except for miR-877, were significantly associated with a poor prognosis.

### 2.6. Functional Enrichment Pathway and Protein–Protein Interaction (PPI) Analysis

The functional enrichment analysis based on KEGG pathways of the six miRNAs showed their involvement in critical cancer signaling pathways, as shown in Table 4. The pathways with a higher number of miRNAs involved (83.3% (5 out of 6)) were thyroid hormone signaling, long-term potentiation, and RNA transport. MiR-1305 was the miRNA involved in all the significant top 10 signaling pathways observed, followed by miR-2116-5p.

**Figure 6:**
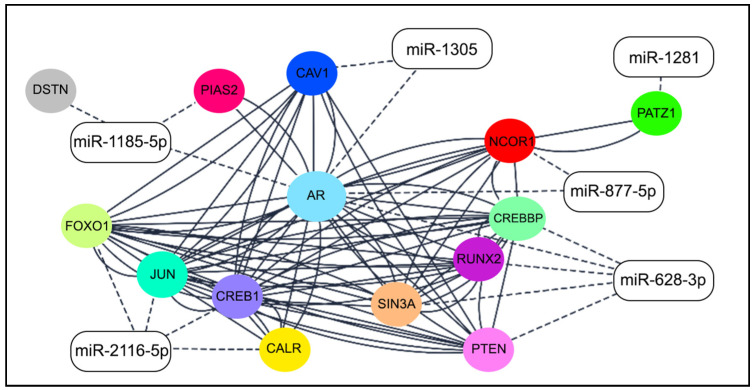
Network of six miRNAs and experimentally validated targets of *AR* gene (color circles). Solid lines: protein–protein interaction; dashed lines: miRNA–mRNA interaction (Cytoscape 3.9.1).

Considering the six miRNAs and their respective target genes involved in AR signaling pathways, their PPI was evaluated using the STRING tool, which showed a network with an average node degree of 5.71, and a local average clustering coefficient of 0.762. The PPI network was directly imported to Cytoscape 3.9.1 to illustrate the interaction among the six miRNAs and their experimentally validated target genes (Figure 6).

## 3. Discussion

AR is a key mediator of androgen signaling, playing significant roles in both normal physiology and cancer. Its involvement in cancer progression, especially in breast cancer, makes AR a critical target for therapeutic intervention [38].

The role of AR in breast cancer is complex and varies among different subtypes. A subset of TNBC expresses AR, and these AR-positive TNBCs might benefit from AR-targeted therapies, presenting a potential new treatment avenue for this aggressive subtype. Conversely, QNBC, which lacks ER, PR, HER2, and AR, cannot be treated by targeting AR, posing a significant treatment challenge [17].

QNBCs represent a distinct subgroup within the broader category of TNBCs. The comparison between QNBCs and TNBCs reveals important differences in their molecular characteristics and clinical outcomes [39]. QNBCs are associated with specific genetic alterations and molecular signatures that distinguish them from other TNBC subtypes, further emphasizing their distinct nature [18]. Patients with QNBCs often experience shorter overall survival and higher rates of disease progression compared to those with TNBCs that express the androgen receptor [39,40]. This highlights the importance of recognizing and studying QNBCs as a separate entity within the broader TNBC category. Understanding the unique characteristics and clinical behavior of QNBCs is crucial for developing targeted therapeutic approaches. Further research is needed to elucidate the underlying mechanisms driving QNBC development and progression, which could potentially lead to the identification of novel therapeutic targets specifically tailored for this subgroup.

The findings of our study revealed a noteworthy disparity in AR loss between AA and EA individuals. Specifically, we observed a higher frequency of AR loss in the AA population compared to the EA population. This observation is consistent with previous studies that have reported racial disparities in AR expression and its related effects on disease outcomes [41,42,43,44]. Furthermore, our study demonstrated a significant association between AR loss and poor prognosis. Patients with AR loss exhibited unfavorable clinical outcomes, including reduced overall survival rates. These findings are in line with the growing body of evidence suggesting the prognostic significance of AR expression in various cancer types, including breast cancer [45,46,47].

The observed racial disparity in AR loss may be attributed to several molecular determinants. Genetic variations, such as single nucleotide polymorphisms (SNPs) or somatic mutations, in genes involved in AR regulation or signaling pathways could contribute to the observed disparity. Additionally, differences in epigenetic modifications, such as DNA methylation or histone modifications, could influence AR expression levels between racial populations [39,48,49,50]. Despite the clear association of AR loss with the AA population in our study, we did not observe significant differences in the DNA methylation profiles of the *AR* gene between the AA and EA QNBC samples of the TCGA dataset. Furthermore, no significant *AR* gene mutations or CNAs were detected in AR-low samples. The lack of differential DNA methylation patterns in the *AR* gene suggests that DNA methylation alterations in the promoter regions may not be the primary driver of AR loss in QNBC cases. While DNA methylation is a well-known mechanism for gene regulation, it appears that other factors, such as epigenetic modifications beyond the promoter regions, could be involved in regulating AR expression and contribute to AR loss in QNBC cases [51,52,53,54]. Moreover, the absence of significant *AR* mutations and CNAs in the AR-low samples suggests that genetic alterations within the *AR* gene itself may not be the primary cause of AR loss in QNBC cases. However, it is important to note that our analysis focused on *AR* mutations and CNAs and may have missed other genetic alterations or regulatory mechanisms affecting AR expression and function. These findings highlight the need for further exploration of alternative mechanisms that contribute to AR loss in QNBC cases. Epigenetic modifications, such as histone modifications or non-promoter DNA methylation, could potentially play a role in regulating AR expression [53,54]. Additionally, the dysregulation of AR-regulating proteins and signaling pathways, including co-regulators or miRNAs, could influence AR expression and function in QNBC [37,55].

Thus, we aimed to investigate the potential alterations in miRNA profiles between racially distinct TNBC and QNBC tumors. MiRNAs have been implicated in various biological processes and are associated with race-related disparities in several diseases, including cancer. By analyzing miRNA expression data from AA and EA breast cancer patients, we sought to identify miRNAs that could potentially contribute to the observed loss of AR expression and racial disparities in disease outcomes. Previous research has suggested that miRNA expression profiles can vary among different racial and ethnic groups, highlighting the need to examine their potential role in health disparities [31,32,33,34,56,57].

In cancer studies, dysregulated levels of miRNAs were shown to interfere in all known hallmarks of cancer, playing important roles in cell proliferation, migration, invasion, angiogenesis, apoptosis, among others [58]. Previous studies conducted in healthy individuals have demonstrated differences in miRNA expression levels between different populations [22,59,60,61,62]. These variants may influence miRNA expression and could explain the differences in the complex traits observed in the populations; however, they do not necessarily impact diseases or cancer risk [62]. In cancer patients with breast cancer, including the TNBC and QNBC subtypes, several studies have also demonstrated that miRNA expression varies in the tumor (somatic) cells in distinct ethnic and racial groups [28,29,31,32,33,63,64,65,66,67,68]. The implication of the differentially expressed miRNAs in these studies lies in the distinctly affected signaling pathways, regulated by the miRNA targets. The distinct involvement of these pathways can directly impact tumor biology, including its aggressiveness, by providing the cells with capabilities for metastasis development and treatment resistance. Therefore, in this study, we hypothesized that differential miRNA expression could also underlie the biological differences between QNBC patients from AA and EA women.

In a discovery cohort of patients with AA QNBCs and EA QNBCs, we found 46 miRNAs that exhibited differential expressions between these groups. The identification of ten differentially expressed miRNAs (miR-1185-5p, miR-1305, miR-3161, miR-3690, miR-494-3p, miR-509-3-5p, miR-619-3p, miR-628-3p, miR-873-5p, and miR-877-5p) directly targeting the *AR* gene underscores their potential role in modulating and regulating AR expression. The analysis in target prediction databases revealed a high score of interaction with these miRNAs and the AR gene. In addition, the analysis of the BRCA TCGA dataset revealed that four of these miRNAs, miR-3161, miR-628-3p, miR-873-5p, and miR-877-5p, presented significant expression correlation with AR expression. These findings offer complementary insights into the regulatory interactions between AR and the miRNAs of our study and align with the intricate regulatory mechanisms associated with miRNAs in gene expression. Moreover, seven of these identified miRNAs were found to target key genes within the AR signaling pathway. Specifically, *AKT1*, *CCND1*, *CDKN1A*, *PTEN*, *RB*, *SIRT1*, and *STAT3* are pivotal components of this pathway, and their modulation by miRNAs suggests a broader impact on the AR-mediated signaling cascade. The regulatory influence of these miRNAs on the *AR* gene and its associated pathway components implies a complex interplay in the context of AA and EA QNBCs. The differential expression of these miRNAs suggests a potential involvement in the molecular mechanisms underlying the observed phenotypic variations between the two ethnic groups.

The examination of differentially expressed (DE) miRNAs in the validation cohort, consisting of 19 QNBC cases (10 AA and 9 EA), provides valuable insights into the molecular landscape of AA and EA QNBC samples. Among the 14 identified DE miRNAs, 6 exhibited consistent expression patterns in both the discovery and validation cohorts, namely miR-1185-5p, miR-1281, miR-1305, miR-2116-5p, miR-628-3p, and miR-877-5p. Remarkably, these six miRNAs demonstrated robust discriminatory power in distinguishing QNBC tumors between AA and EA women. The AUC values, ranging from 0.7576 to 1, underscored their effectiveness in capturing the molecular distinctions between these ethnic groups. This aligns with the notion that miRNA expression profiles can serve as reliable biomarkers for QNBC subtypes across diverse populations.

Among these miRNAs, miR-1185 was shown to be down-regulated in colorectal cancer cells in association with the mTOR pathway [69]. Contrary to our findings in the QNBC patients, this study suggests a tumor suppressor role for this miRNA. MiR-1281 expression was described as down-regulated in osteosarcoma and bladder cancer patients [70,71]. In the osteosarcoma study [70], when apoptosis was induced by ER stress, an increase in miR-1281 expression was observed. On the other hand, the inhibition of this miRNA resulted in reduced tumor growth and an increase in cell apoptosis. Other studies in colorectal and gastric cancer and gliomas have shown that long non-coding RNAs (lncRNAs) regulate the expression of miR-1281, leading to an increase in tumorigenesis [72,73,74] and aggressive phenotypes. In our study, this miRNA was observed to be up-regulated in both the discovery and validation cohorts, suggesting that it might present a different mode of action in breast cancer. MiR-1305 is prominently involved in numerous cancer-related signaling pathways in our study. Notably, it has been recognized as a tumor suppressor in liver cancer [75], where it also plays a role in influencing the stemness of cancer cells. In non-small cell lung cancer [76], a correlation between lower levels of miR-1305 and metastasis and poor prognosis of the patients was observed. Its overexpression, on the other hand, resulted in the inhibition of proliferation and migration and promoted apoptosis. These findings, together with our own, also pointed out a tumor suppressor role for miR-1305. Unlike the other miRNAs, miR-2116-5p has not been extensively reported as having a role in cancer cells. In fact, the limited data available are related to miR-2116-3p, which was found with potential tumor-inhibiting action in breast [77] and colorectal [78] cancer, and glioblastoma [79]. MiR-628-5p, on the other hand, was extensively reported with deregulated expression in cancer cells. Contrary to our study, which demonstrated up-regulated levels of this miRNA in the QNBC cells, in TNBC, it was shown to suppress proliferation and metastasis. This suppression occurred by negatively regulating SMAD3 expression [80]. Finally, miR-877-5p, the only miRNA that did not show significant correlation with survival in the TCGA and METABRIC breast cancer cases, was indicated as a potential prognostic marker for the early detection of tumor development and progression, as well as metastasis in several types of cancer, including breast cancer [81]. In agreement with most of the cancer studies reported, this miRNA presents a tumor suppressor action and was found to be down-regulated in tumor cells compared to adjacent non-tumor tissue. Functional studies have shown its role in suppressing malignancy through its involvement in several pathways in cancer, where it mediates the regulation of critical genes, such as *CDK4* and *FOXM1* [82,83,84,85].

Notably, the observed miRNAs also presented a role in influencing the tumor microenvironment (TME), as characterized by changes in cell–cell interactions, structural support provided by the extracellular matrix (ECM), and the composition of the stromal cells and their releasing signal factors, as well as conditions of oxygen deprivation (hypoxia) and nutrient scarcity [86,87,88,89]. Among the six miRNAs identified, miR-1305 is the one most reported to be associated with TME modulation. In ovarian cancer cells, miR-1305 was reported to regulate dormancy linked to the IL-6 soluble factor by controlling the expression of the *ARH-I* gene [90], a tumor suppressor gene known for inducing autophagy in a nutrient deprivation environment [91]. Interestingly, miR-1305 overexpression was observed in exosomes of hypoxic multiple myeloma cells, contributing to tumorigenic effects in macrophages and emphasizing its role in TME modulation [92]. MiR-2116-5p was shown to indirectly impact the TME in breast cancer cells by being sponged by the long non-coding RNA LINC01433, activating the *MYC* oncogene [93], a potent TME regulator [77]. Similarly, in pancreatic cancer, hypoxia-induced exosomal circPDK1, which regulates miR-628-3p, promotes glycolysis via *MYC* activation [94]. Furthermore, miR-628-5p exosomes derived from M1 macrophages inhibited human methyltransferase-like 14 (*METTL14*) expression in hepatocellular cancer, influencing the expression of circFUT8 and suppressing tumor progression [95]. Collectively, these studies underscore the involvement of the identified miRNAs in TME regulation, acting through diverse mechanisms in response to cellular factors and TME signals.

The signaling pathways mostly affected by these six miRNAs include the thyroid hormone signaling Hippo signaling pathway, signaling pathways regulating the pluripotency of stem cells, FOXO, gap junction, and RNA transport. While the AR signaling pathway is distinct from these observed signaling pathways, crosstalk and interconnections among these pathways have been described. For instance, thyroid hormone receptors were shown to modulate AR signaling, potentially influencing AR-mediated gene expression and cellular processes [96]. The signaling pathways regulating the pluripotency of stem cells, which among several pathways include the Wnt and Hippo pathways, may influence cellular differentiation processes that intersect with AR-regulated cellular programs. Studies in prostate cancer have shown that the AR and YAP (Hippo pathway effector Yes-associated protein (YAP) interact and that their translation and activity are correlated [97,98,99,100]). It is important to note however that the specific nature and extent of these interconnections may vary depending on the cell type, tissue context, and specific cellular processes under investigation. Further research is needed to fully elucidate the mechanisms and functional consequences of these interactions with the AR pathway and develop targeted therapies. Developing therapeutic agents and delivery systems, using miRNA mimic and/or inhibitor (antagomiRs) strategies, can restore the function of down-regulated miRNAs or block the function of up-regulated miRNAs, respectively. These approaches have the potential to regulate AR signaling, which may play a role in QNBC progression, particularly in AA patients who exhibit distinct miRNA expression patterns. Such targeted therapies could help refine treatment options for this aggressive breast cancer subtype. Additionally, AR modulators, such as AR antagonists or selective androgen receptor degraders (SARDs), may serve as another promising therapeutic strategy. Tailoring treatment regimens based on miRNA expression profiles and AR status can lead to more effective and personalized therapeutic approaches, ultimately improving outcomes, especially for patients of African descent.

In summary, we report the differential expression of miRNAs between AAs and EAs with QNBC, including miRNAs predicted to target the AR gene/signaling. However, a significant limitation of this study is the lack of functional validation experiments to confirm the predicted miRNA–AR interactions. While we utilized computational databases to identify potential miRNA targets and construct an interaction network with high interaction scores, these predictions alone do not provide direct evidence of functional regulatory mechanisms. Future research employing functional assays, such as luciferase reporter and miRNA transfection mimic/inhibition systems, would provide critical insights into the direct interactions between these miRNAs and AR. Conducting these additional studies will be essential to advancing the understanding of miRNA–AR regulatory dynamics in QNBC. Another key limitation of this study is the relatively small sample size of the validation cohort. Although the identified miRNA profiles showed promising discriminatory power, the limited sample size may reduce the robustness of our results and may not fully capture the diversity within the broader QNBC patient population. Future studies should aim to validate these findings in larger and more diverse clinical cohorts to confirm the consistency of miRNA expression patterns across various demographic and clinical subgroups.

## 4. Materials and Methods

### 4.1. Study Design

The general workflow of this study is presented in Figure S2. In addition, we assessed the race-annotated TNBC cases for AR expression and further classified them as TNBC (AA and EA) and QNBC (AA and EA). Tumors from all four groups were isolated for RNA and profiled for global miRNA expression. Functional enrichment pathway analysis was performed to understand how the chosen miRNAs and their associated mRNA targets influence biological functions related to the AA and EA QNBC phenotype. Additionally, we explored protein–protein interaction (PPI) to construct a network illustrating the pairings between miRNAs and mRNAs. Receiver Operating Characteristic (ROC) analysis was used to determine the discriminatory power of the selected miRNAs in differentiating TNBCs and QNBCs. Finally, the miRNA expression data were associated with the clinicopathologic and follow-up information from the patients.

### 4.2. Study Cohort

A total of 129 TNBC cases (60.5% of AA and 39.5% of EA patients) were identified from the Emory Decatur Hospital (n = 78), Atlanta, GA, and Histopathology and Tissue Shared Resources (HTSR) of Lombardi Comprehensive Cancer Center (LCCC), Georgetown University (n = 51), Washington DC, USA. The cases collected were treatment-naïve at the time of surgery. Formalin-fixed paraffin-embedded (FFPE) samples were obtained for this study along with the clinical–histopathological data and outcomes. All the aspects of the study including protocols, sample procurement, and whole study design were approved by Institutional Review Boards (IRB#1992-048 and #H19306). The FFPE specimens were received de-codified, with no patient identifiers. The clinicopathological characteristics were reviewed and provided by the respective hospitals, and the average time of follow-up from the patients was 4.789 ± 3.094 years. No significant differences were observed among the variables analyzed. The clinical–pathological parameters and follow-up data of the AA and EA patients are provided in Table 5.

### 4.3. Immunohistochemistry (IHC)

The FFPE sections of the 129 cases were stained for AR, as previously described [17]. Firstly, the tissue sections were deparaffinized using xylene, followed by rehydration through a series of ethanol washes at various concentrations (100%, 90%, 75%, and 50%). To retrieve antigens, a pressure cooker was used with a citrate buffer (pH 6.0) at 15 psi for 30 min. Hydrogen peroxide was applied for 20 min to quench the samples, which were then subjected to a series of buffer washes. Next, the tissues were blocked using the ultra-vision protein block (Life Sciences, Fremont, CA, USA) for 10 min, followed by a 60 min incubation with an anti-AR primary antibody (Monoclonal Mouse Anti-Human Androgen Receptor, clone AR 441, Dako North America Inc., Carpinteria, CA, USA) diluted at 1:40, at room temperature. After further washes, the samples were incubated with MACH2 HRP-conjugated secondary antibody (BioCare Medical, Pacheco, CA, USA) for 30 min. For enzymatic antibody detection, the tissues were exposed to the Betazoid DAB Chromogen Kit (BioCare Medical, Pacheco, CA, USA). Finally, Mayer’s hematoxylin was applied as a counterstain for 1 min, followed by dehydration in alcohol, clearing in Xylene, and mounting with mounting media. The slides were scored for AR by two independent pathologists without prior knowledge of the patients’ pathologic or outcome data. AR expression in more than 1 percent of the cells was considered positive for AR while TNBC samples were considered quadruple-negative (QNBC) if they were present in less than 1 percent of the cells positive for AR.

### 4.4. AR Gene Methylation Status, Mutations, and Copy Number Alterations Analysis (TCGA Database)

The genomic profile of AR in the TCGA breast invasive carcinoma (BRCA) dataset was obtained from cBioPortal (https://www.cbioportal.org/ (accessed on 7 October 2024)). First, the TCGA sample IDs corresponding to AR-low BRCA samples were pasted in the “Select Patient/Case Set” text box and then the database was queried for the mutation and copy number alteration profile of AR in the selected samples. Oncoprints depicting a proportion of the samples with alteration were downloaded. DNA methylation data related to TCGA BRCA dataset generated on Illumina Infinium HumanMethylation450 BeadChip was downloaded using TCGA-assembler pipeline [101]. The downloaded data includes the beta value for all CpG probe IDs for all BRCA samples considered (n = 793). The beta values ranged from 0 to 1 (representing no methylation to complete methylation). Using built-in functions of the TCGA-assembler, the single methylation value for the promoter region (−1500 to +1) of each gene was calculated in each sample.

### 4.5. RNA Isolation

Firstly, the cases were evaluated for the total tumor area in an H&E slide and only the cases with at least an 80% tumor area were considered for RNA isolation. The tumor areas were selected in the 5 µm unstained FFPEs with reference to H&E, microdissected, and the RNA was isolated using TRIzol (Invitrogen Carlsbad, CA, USA). The NanoDropTM Spectrophotometer (Thermo Scientific Inc., Waltham MA, USA) and the Bioanalyzer (Agilent Technologies Inc., Santa Clara, CA, USA) were used to assess the quantity and quality of the isolated RNA, respectively. From all the cases assessed for the content of the tumor cells and RNA quality, 62 cases were selected for miRNA expression profiling based on these criteria.

### 4.6. MiRNA Expression Analysis

The 61 cases were divided into 2 cohorts in a proportion of about 70% (discovery cohort (n = 42)) to 30% (validation cohort (n = 19)). In the discovery cohort, the AA QNBCs (n = 33) and EA QNBCs (n = 9) cases profiled for miRNA were from the Emory Decatur Hospital and HTSR of LCCC, Georgetown University (n = 51), and in the validation cohort, the AA QNBCs (n = 10) and EA QNBCs (n = 9) cases were from HTSR of LCCC, Georgetown University. MiRNA expression analysis was performed in these cohorts using Nanostring nCounter Human v3a miRNA Expression Assay (Seattle, WA, USA), as previously performed [32,33,37]. This platform contains human probes derived from miRBase version 22 (https://www.mirbase.org (accessed on 3 April 2024)), targeting 827 human miRNAs, 6 positive controls, 8 negative controls, 3 ligation positive controls, 3 ligation negative controls, 5 internal reference genes (*ACTB*, *B2M*, *GAPDH*, *RPL19*, and *RPL0*), and 5 spike-in controls (ath-miR-159a, cel-miR-248, cel-miR-254, osa-miR-414, and osa-miR-442). Raw data were pre-processed using the Nanostring’s Counter RCC collector and normalized using Nanostring nSolver 4.0 software. Specifically, each RCC file was uploaded, the background was subtracted (negative control geometric mean), and the data were normalized (positive control normalization: geometric mean; and CodeSet Content normalization to all genes, geometric mean). The normalized data were Log2 transformed and analyzed using the MultiExperiment Viewer software (MeV 4.9.0) as described. The unsupervised (UHC) and supervised hierarchical cluster (SHC) analysis were performed on significantly differentially expressed miRNAs among the patients’ subtypes (defined as presenting fold changes (FC) > 1.5 (log2FC > ±0.58), using Pearson correlation coefficient, average linkage, and Benjamini–Hochberg multiple testing correction (*t*-test *p* < 0.01, FDR < 0.05).

### 4.7. MiRNA Targets Selection

Gene targets were queried using the available miRNA target databases (Diana micro-T-CDS v.5.0 (diana.imis.athena-innovation/gr/DianaTools (accessed on 3 April 2024)), miRDB (http://www.mirdb.org/miRDB/ (accessed on 3 April 2024)) and TargetScan Release 7.1 (http://www.targetscan.org/vert_71/ (accessed on 3 April 2024)); only the miRNA target genes that were present in two out of the three miRNA databases were selected. Targets that were directly related to the *AR* gene or AR signaling pathway were also identified in the above databases. The WikiPathways open-collaborative platform was used to search for related androgen receptor signaling pathway genes.

### 4.8. Biological Function and Pathway Analysis

To assess how the dysregulated miRNAs identified in the analyzed groups may influence cancer-related biological processes and pathways, we employed Diana miRPath v.3.0 (diana.imis.athena-innovation/gr/DianaTools (accessed on 3 April 2024)). The analysis considered adjusted *p*-values with FDR correction. Enrichment analysis of multiple miRNA gene targets comparing each set of miRNA targets to all known KEGG (Kyoto Encyclopedia of Genes and Genomes) pathways were obtained and selected by significant *p*-value (*p* < 0.05) and cancer-associated biological functions.

### 4.9. Interaction of miRNA–mRNA Pairings

The databases miRTarBase v.9.0 and Tarbase v.8.0 [102,103] were used to determine interactions between the selected miRNAs and target genes, which were validated based on strong (reporter assays, Western blot, and qPCR) and less strong experimental assays (microarray, NGS, pSilac). The STRING v.11.5 database (https://www.string-db.org (accessed on 24 April 2024)) was used to verify protein–protein interaction (PPI) between the validated target genes of each group, applying the following parameters: connections based on co-expression, co-expression-transferred, database, database-transferred, experiments, and experiments-transferred, and a minimum interaction score of 0.9 (highest confidence). Cytoscape v.3.9.1 (https://cytoscape.org (accessed on 24 April 2024)) was used to construct the molecular interaction networks of the selected miRNAs and target genes. In addition, the TCGA BRCA dataset was conducted to investigate the relationship between AR expression (RNA-seq) and the expression of the ten miRNAs (miRNA-seq) that were predicted to target AR. This analysis was performed both in the TNBC and BRCA dataset of the TCGA. Pearson correlation was performed to determine the correlation of the miRNA and AR expression.

### 4.10. Statistical Analysis

The ROC analysis was used to calculate the area under the curve (AUC) to identify the discriminatory power of the selected miRNAs in differentiating the TNBC from the QNBC cases. Sensitivity was plotted against 1-specificity for the binary classifier (TNBC and QNBC). AUCs and 95% corresponding confidence intervals were calculated for each miRNA and the combined miRNAs. The correlation of the selected miRNAs expression levels with the patients’ clinical–pathological parameters and follow-up data were performed using Pearson correlation. The GraphPad Prism 8.3.0 was used to perform the analyses. Kaplan–Meier plotter (https://kmplot.com (accessed on 1 May 2024)) was used to assess the correlation between the miRNA expression levels and overall survival of TNBC patients from the METABRIC and TCGA databases. Log-rank *p* < 0.05 was considered as significant.

## 5. Conclusions

Our study highlights a notable racial disparity in AR loss, with a higher frequency observed in AA TNBCs compared to the EAs. The study found no significant DNA methylation or *AR* gene mutations, suggesting alternative mechanisms at play. Multiple miRNAs, targeting AR and AR-associated pathways, displayed varying expression profiles in the QNBC samples from AA and EA women. It is critical to point out however that there might be other epigenetic alterations that lead to AR loss in QNBC, other than the ones evaluated in this study. Long non-coding RNAs (lncRNAs) and other miRNAs, for example, could also contribute to AR expression regulation in QNBC, interacting with the AR gene or regulating chromatin states associated with AR expression. It is also important to mention however that the difference in the frequency of AR loss observed in the AA and EA TNBC patients may also be influenced by non-biological factors, including socioeconomic status, healthcare accessibility, and environmental exposure. These elements contribute to differences in both disease outcomes and responses to treatment. A comprehensive approach is necessary to address and alleviate these racial disparities in breast cancer. In this sense, our study emphasizes the need for an in-depth exploration of miRNA involvement and the complex interactions among AR-associated signaling pathways and epigenetic modifications in QNBCs of distinct racial and ethnic cases, while considering the mentioned confounding factors. Understanding these mechanisms is crucial for tailored treatments and improved outcomes in patients that suffer from breast cancer disparities.

## Figures and Tables

**Table 1:** The fold change (FC) and *p*-values of the 10 miRNAs within the 46 DE miRNAs in the QNBC tumors of the AA and EA patients that are predicted to target the *AR* gene.

	Log2FC	*p*-Value
hsa-miR-1185-5p	1.012128	0.037838
hsa-miR-1305	−0.999874	0.007647
hsa-miR-3161	−0.570746	0.038412
hsa-miR-3690	0.216897	0.047954
hsa-miR-494-3p	−0.943088	0.024355
hsa-miR-509-3-5p	0.265410	0.020465
hsa-miR-619-3p	0.259912	0.030826
hsa-miR-628-3p	0.455397	0.001020
hsa-miR-873-5p	0.364058	0.024755
hsa-miR-877-5p	−1.080339	0.0168850

**Table 2:** Seven miRNAs, within 46 DE miRNAs in QNBC tumors of AA and EA patients, with experimentally validated targets on AR signaling pathway (mirPathDB-AR signaling, *p*-value < 0.05).

miRNA	Evidence	*p*-Value	Targets
hsa-miR-1258	Experimental (any)	0.038	*RB1*, *RLN1*
hsa-miR-184	Experimental (any)	0.045	*AKT1*, *CARM1*
hsa-miR-429	Experimental (any)	0.006	*EP300*, *JUN*, *NCOA2*, *PTEN*, *SP1*
hsa-miR-494-3p	Experimental (any)	8.72 × 10^−4^	*AKT1*, *BAG1*, *CCND1*, *PTEN*, *RB1*, *RHOB*
hsa-miR-520c-3p	Experimental (strong)	0.025	*SIRT1*, *STAT3*
hsa-miR-628-3p	Experimental (any)	0.031	*CREBBP*, *RUNX2*
hsa-miR-95-3p	Experimental (strong)	0.007	*CCND1*, *CDKN1A*

**Table 3:** Fourteen common miRNAs between discovery and validation cohorts of QNBC patients.

miRNAs	Discovery Set	Validation Set
hsa-miR-1185-5p *	Up-regulated	Up-regulated
hsa-miR-1200	Up-regulated	Down-regulated
hsa-miR-1244	Up-regulated	Down-regulated
hsa-miR-1258 **	Up-regulated	Down-regulated
hsa-miR-1281	Up-regulated	Up-regulated
hsa-miR-1293	Up-regulated	Down-regulated
hsa-miR-1305 *	Down-regulated	Down-regulated
hsa-miR-2116-5p	Down-regulated	Down-regulated
hsa-miR-218-5p	Down-regulated	Up-regulated
hsa-miR-608	Up-regulated	Down-regulated
hsa-miR-613	Up-regulated	Down-regulated
hsa-miR-628-3p *,**	Up-regulated	Up-regulated
hsa-miR-873-5p *	Up-regulated	Down-regulated
hsa-miR-877-5p *	Down-regulated	Down-regulated

* miRNA predicted to directly target *AR* gene, ** miRNAs predicted to target AR signaling pathway.

**Table 4:** Top ten KEGG pathways, with corresponding *p*-value, number of gene targets and miRNAs, of six miRNAs with high power in discriminating AA and EA QNBCs (presented by *p*-value).

KEGG Pathways	*p*-Value	# Genes	# miRNAs	miRNAs
1. Hippo signaling pathway	6.14 × 10^−5^	32	4	miR-1305, miR-628-3p, miR-1185-5p, miR-877-5p
2. Thyroid hormone signaling pathway	0.001291	32	5	miR-1305, miR-2116-5p, miR-1185-5p, miR-628-3p, miR-877-5p
3. Mucin type O-Glycan biosynthesis	0.001804	7	1	miR-1305
4. TGF-beta signaling pathway	0.002344	19	2	miR-1305, miR-2116-5p
5. Signaling pathways regulating pluripotency of stem cells	0.002344	35	4	miR-1305, miR-877-5p, miR-628-3p, miR-2116-5p
6. FoxO signaling pathway	0.002454	35	4	miR-628-3p, miR-1185-5p, miR-1305, miR-2116-5p
7. Gap junction	0.009118	21	4	miR-1305, miR-2116-5p, miR-628-3p, miR-877-5p
8. Long-term potentiation	0.014921	20	5	miR-1305, miR-1185-5p, miR-628-3p, miR-877-5p, miR-2116-5p
9. RNA transport	0.01845	37	5	miR-1305, miR-1185-5p, miR-628-3p, miR-877-5p, miR-2116-5p
10. Dopaminergic synapse	0.036055	30	4	miR-1185-5p, miR-1305, miR-2116-5p, miR-628-5p

**Table 5:** Clinical–pathological parameters and follow-up data of 129 TNBC patients studied, distributed by race.

	AA	EA	*p*-Value
Age (yrs)	49.814 ± 11.120	52.523 ± 13.741	*p* = 0.1326
	(n = 74)	(n = 48)	
Tumor size (cm)	3.380 ± 2.910	3.023 ± 6.516	*p* = 0.3583
	(n = 73)	(n = 44)	
Tumor grade			
1 + 2	11	5	*p* ≥ 0.05
3	63	40	
	(n = 74)	(n = 45)	
Ki67			
≤14 (low)	4	2	*p* ≥ 0.05
>14 (high)	41	21	
	(n = 45)	(n = 23)	
Vital status			
Alive	40	31	*p* ≥ 0.05
Deceased	37	21	
	(n = 77)	(n = 52)	

## Data Availability

The data underlying this article will be shared on request to the corresponding author.

## Supplementary Materials

The following supporting information can be downloaded at: https://www.mdpi.com/article/10.3390/ijms252413679/s1.
