# Supplementary material for: Dysregulated miRNA Expression and Androgen Receptor Loss in Racially Distinct Triple-Negative Breast Cancer
Source: Int J Mol Sci. 2024 Dec 21;25(24):13679. doi: 10.3390/ijms252413679 (PMC11679545; doi:10.3390/ijms252413679)
Supplement: Supplementary file 1 [file ijms-25-13679-s001.zip › ijms-3273181-supplementary.pdf]

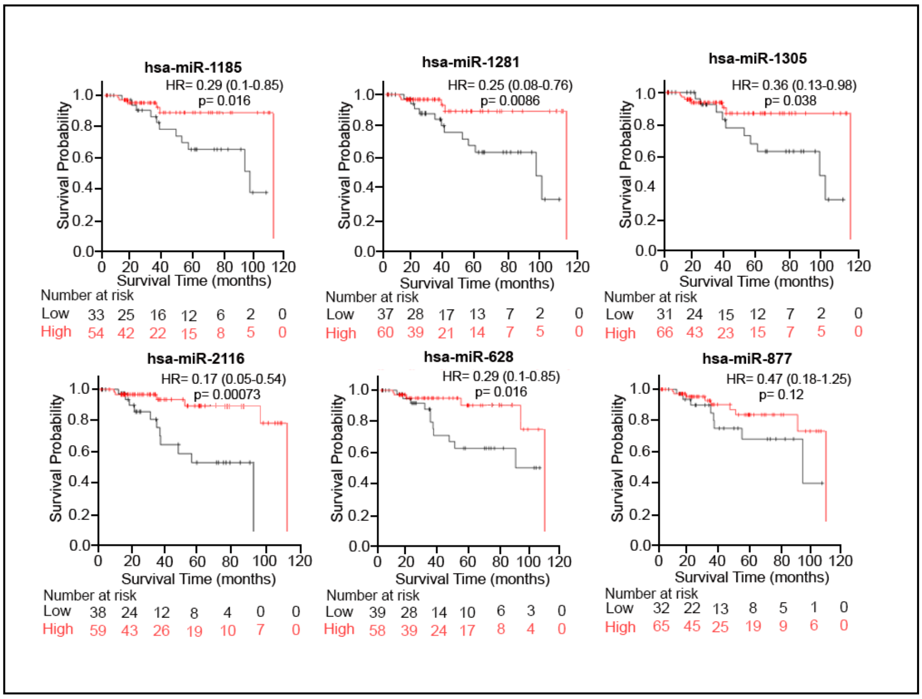


**Figure S1:** Survival analysis of patients with TNBC of the TCGA and METABRIC cohorts. Significance $p < 0.05$.

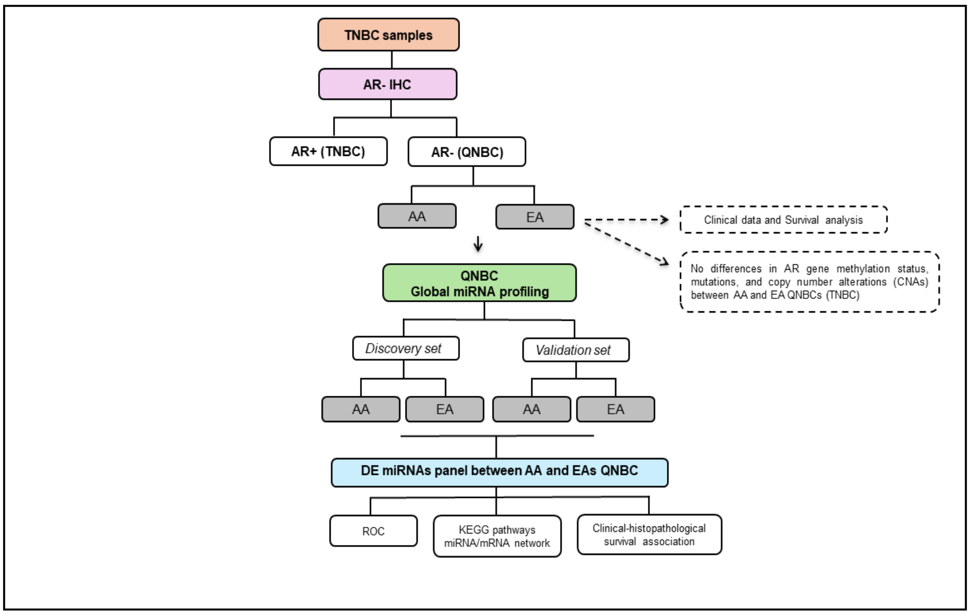


**Figure S2.** Study design.

**Table S1.** Forty-six differentially expressed miRNAs between AA and EA QNBC patients (global miRNA profiling analysis).

| miRNA | Log2FC | P value | FDR |
|---|---|---|---|
| hsa-miR-513a-5p | 0.649637485 | 0.000486798 | 0.38846502 |
| hsa-miR-628-3p | 0.45539681 | 0.001019636 | 0.40683454 |
| hsa-miR-1275 | 0.56445268 | 0.001639689 | 0.43615717 |
| hsa-miR-188-5p | 140.054.591 | 0.002386734 | 0.47615346 |
| hsa-miR-142-3p | 116.649.636 | 0.002960973 | 0.47257137 |
| hsa-miR-585-3p | -1.054.065 | 0.002987207 | 0.39729849 |
| hsa-miR-98-3p | 0.35388801 | 0.003497878 | 0.3987581 |
| hsa-miR-582-5p | -0.88928858 | 0.003628842 | 0.361977 |
| hsa-miR-767-3p | 0.540445172 | 0.005242269 | 0.46481448 |
| hsa-miR-429 | 14.870.755 | 0.005277272 | 0.4211263 |
| hsa-miR-608 | 0.37460403 | 0.0068422 | 0.49637052 |
| hsa-miR-218-5p | -130.916.292 | 0.007001441 | 0.46559584 |
| hsa-miR-1305 | -0.99987418 | 0.007647317 | 0.46942765 |
| hsa-miR-1244 | 0.368875709 | 0.008183889 | 0.4664817 |
| hsa-miR-1200 | 126.671.245 | 0.008663866 | 0.46091768 |
| hsa-miR-412-3p | 0.305351277 | 0.01319179 | 0.65794057 |
| hsa-miR-1281 | 0.42775466 | 0.014021234 | 0.6581732 |
| hsa-miR-1258 | 0.33039988 | 0.014047263 | 0.622762 |
| hsa-miR-499b-3p | 0.350417997 | 0.0152116 | 0.63888717 |
| hsa-miR-1266-5p | 0.251525915 | 0.015397009 | 0.61434066 |
| hsa-miR-877-5p | -108.033.928 | 0.016884927 | 0.6416272 |
| hsa-miR-1304-5p | 0.325835321 | 0.01690726 | 0.6132724 |
| hsa-miR-620 | 0.247487047 | 0.019266639 | 0.6684686 |
| hsa-miR-509-3-5p | 0.26541036 | 0.0204646 | 0.6804479 |
| hsa-miR-613 | 0.95076234 | 0.022046968 | 0.7037392 |
| hsa-miR-95-3p | -0.70681107 | 0.022148497 | 0.67978853 |
| hsa-miR-3168 | 103.967.501 | 0.023507448 | 0.69477564 |
| hsa-miR-184 | 0.5904705 | 0.023521928 | 0.67037493 |
| hsa-miR-519b-5p | 0.319540392 | 0.023959115 | 0.65928876 |
| hsa-mir-519c-5p | 0.319540392 | 0.023959115 | 0.65928876 |
| hsa-miR-523-5p | 0.319540392 | 0.023959115 | 0.65928876 |
| hsa-miR-518e-5p | 0.319540392 | 0.023959115 | 0.65928876 |
| hsa-miR-522-5p | 0.319540392 | 0.023959115 | 0.65928876 |
| hsa-miR-494-3p | -0.9430876 | 0.024354964 | 0.6478421 |
| hsa-miR-873-5p | 0.364058297 | 0.02475513 | 0.63724494 |
| hsa-miR-619-3p | 0.259912455 | 0.030825777 | 0.7687178 |
| hsa-miR-214-3p | -15.454.635 | 0.034695134 | 0.83899146 |

| | | | |
|---|---|---|---|
| hsa-miR-2116-5p | -0.89347726 | 0.035609983 | 0.83578724 |
| hsa-miR-1293 | 0.260347897 | 0.036682907 | 0.8363703 |
| hsa-miR-369-5p | 0.199179125 | 0.037192125 | 0.82442546 |
| hsa-miR-1185-5p | 1.012.128.168 | 0.03783817 | 0.8160773 |
| hsa-miR-520c-3p | 0.230255287 | 0.037851885 | 0.79488957 |
| hsa-miR-3161 | -0.57074585 | 0.03841242 | 0.7859772 |
| hsa-miR-584-5p | -0.93575853 | 0.043783214 | 0.87347513 |
| hsa-miR-3690 | 0.216897227 | 0.047954462 | 0.93335754 |
| hsa-miR-1973 | -0.76343903 | 0.04930853 | 0.9368621 |
